# Explainable AI for Equitable Nurse Scheduling: Pragmatic Pre-Post Implementation Study

**DOI:** 10.2196/94450

**Published:** 2026-07-02

**Authors:** Ben-Chang Shia, Szu-Ming Peng, Qui-Yang Zhang, Chiung-Yun Lo, Sheng-Ru Wang

**Affiliations:** 1 Graduate Institute of Business Administration, College of Management, Fu Jen Catholic University, New Taipei City, Taiwan; 2 Deputy Director of Teaching and Research Department, St. Paul’s Hospital, Taoyuan City, Taiwan; 3 Department of Pediatric Emergency, Fu Jen Catholic University Hospital, No. 69, Sec. 1, Gui-zi Road, Taishan Dist., New Taipei City, 24352, Taiwan, 886 0926189605

**Keywords:** nurse scheduling, workload equity, explainable artificial intelligence, algorithmic fairness, implementation science, decision support systems, workforce management

## Abstract

**Background:**

Inequitable and time-consuming shift scheduling contributes to nurse burnout, dissatisfaction, and turnover. In Taiwan, annual nurse turnover reaches 11.6%, with rigid 3-shift systems and unfair workload distribution frequently cited as key drivers. Although artificial intelligence (AI) scheduling tools exist, most lack transparency and do not formally address algorithmic bias, limiting clinical adoption.

**Objective:**

This study aimed to design, deploy, and evaluate a transparent, fairness-audited, explainable AI–enabled nurse scheduling decision support system (XAI-NSDSS) to reduce administrative burden, eliminate experience-based algorithmic bias, and enhance staff acceptance in a real-world hospital setting.

**Methods:**

A pragmatic before-after implementation study was conducted at a 671-bed teaching hospital in Taiwan (January-December 2023), involving 8 departments and 156 nurses (42 novice, 78 midlevel, and 36 experienced). A 6-month manual scheduling baseline (January-June 2023) was compared with a 6-month AI-assisted period (July-December 2023). The XAI-NSDSS integrates a random forest workload prediction model (*R*²=0.887), Shapley Additive Explanations–based explainability, a hybrid integer programming and binary differential evolution (IP+ BDE) optimizer, and a multidimensional fairness monitoring dashboard. A formal weight sensitivity analysis (WSA) was conducted across 7 prespecified weight configurations using full-factorial repeated-measures ANOVA to assess outcome robustness. Primary outcomes were scheduling time, error rate, and user satisfaction. Statistical analyses used linear mixed effects models (LMMs) and generalized estimating equations (GEE) with department as a random effect.

**Results:**

Monthly scheduling time decreased by 81.2% (mean 32.0, SD 8.0-mean 6.0, SD 2.0) hours; *P*<.001; Cohen *d*=4.33) and error rate decreased by 73.8% (mean 18.3, SD 4.3%-mean 4.8, SD 1.2%; *P*<.001; Cohen *d*=4.12). Nurse satisfaction improved from a mean of 3.2 (SD 0.8) to a mean of 4.4 (SD 0.6; *P*<.001), with 148 out of 156 nurses (94.9%) adopting the system by Month 3. Preexisting experience-based bias was fully eliminated: workload coefficient of variation (CV) decreased 50% (0.18-0.09; *P*<.001), disparate impact ratios normalized from 1.35‐1.56 to 1.01‐1.04, and preference satisfaction equity was achieved across experience tiers (ANOVA *P*=.38). Among 156 nurses, 82 (52.6%) regularly engaged with Shapley Additive Explanations; this engagement was positively associated with satisfaction (Pearson *r*=0.456; *P*<.001). The WSA across 7 configurations confirmed that the consensus-derived default weights achieved the highest composite quality score (mean 82.1, SD 3.2) and that disparate impact ratios remained within the 0.80‐1.25 fairness threshold across all configurations (*P*=.12), demonstrating structural robustness of the fairness-auditing module.

**Conclusions:**

This study presents the first longitudinally validated explainable AI implementation framework for nurse scheduling with formal algorithmic fairness auditing and WSA. The XAI-NSDSS framework is replicable, scalable, and provides a practical blueprint for responsible AI adoption in health care workforce governance, with fairness guarantees that are robust to institutional customization of optimization priorities.

## Introduction

### Background and Rationale

The global nursing workforce crisis is one of the most pressing challenges in modern health care. The World Health Organization (WHO) projects a shortage of 5.9 million nurses by 2030, with high-income countries experiencing turnover exceeding 15% annually [1]. In Taiwan, the annual nursing turnover rate reached a 10-year high of 11.6%, with scheduling-related dissatisfaction identified as a leading contributor to burnout and early career exit [2,3]. Inefficient scheduling creates a vicious cycle; unfair shift distribution increases fatigue, which accelerates attrition, which further strains remaining staff.

Nurse scheduling is a complex combinatorial optimization problem requiring simultaneous consideration of regulatory constraints (labor laws and union agreements), operational requirements (shift coverage and skill mix), and individual staff preferences (time-off requests and work-life balance) [4]. Traditional manual scheduling is time-intensive, error-prone, and chronically fails to achieve fair workload distribution, particularly disadvantaging novice nurses who lack institutional leverage to negotiate favorable assignments [5].

### Gaps in Existing AI Scheduling Systems

Artificial intelligence (AI) and operations research approaches have shown promise in nurse scheduling. Recent implementations include mixed integer programming heuristics [6], genetic algorithms [7], and machine learning–based prediction models [8]. However, a systematic review of the literature reveals four persistent, unresolved gaps:

1. Opacity and lack of explainability: most systems operate as “black boxes,” generating schedules without interpretable rationale, a critical barrier to trust in high-accountability health care environments [9,10].
2. Absence of formal fairness auditing: while scheduling optimization papers frequently mention equity as an objective, formal auditing of algorithmic bias using established fairness metrics (eg, disparate impact ratios) remains virtually absent from the literature [11].
3. Insufficient real-world longitudinal validation: the majority of published systems are evaluated on simulated or retrospective datasets. Prospective, hospital-based evaluations are rare, limiting evidence for practical adoption [12,13].
4. Neglect of user trust mechanisms: system design rarely accounts for how transparency features influence actual adoption behavior, creating a gap between algorithmic performance and clinical uptake [14,15].

### Study Objectives and Contributions

This study directly addresses all 4 gaps through the design, deployment, and 12-month evaluation (6-mo pre- and 6-mo postimplementation) of the explainable AI–enabled nurse scheduling decision support system (XAI-NSDSS) at a 671-bed tertiary referral hospital in Taiwan. The aim of this study was to demonstrate that a single integrated framework can simultaneously achieve operational efficiency, provable algorithmic equity, and clinician trust through explainability. Specific contributions are as follows:

1. Framework contribution: among the first end-to-end XAI implementation frameworks for nurse scheduling integrating Shapley Additive Explanations (SHAP)–based explainability, formal optimization, and a multidimensional algorithmic fairness monitoring system.
2. Empirical contribution: prospective real-world evidence from 156 nurses across 8 departments over 12 months, among the largest single-site longitudinal evaluations of an explainable artificial intelligence (XAI) nurse scheduling system reported to date.
3. Fairness contribution: formal documentation and complete elimination of preexisting experience-based algorithmic bias using disparate impact ratios, Gini coefficients, and ANOVA-based equity testing.
4. Trust mechanism contribution: empirical evidence that SHAP engagement (82/156, 52.6% nurses) is positively associated with satisfaction (*r*=0.456; *P*<.001), positioning explainability as a functional organizational intervention.
5. Implementation science contribution: actionable guidelines for change management, governance, and phased rollout based on observed adoption patterns, learning curves, and organizational barriers.

## Methods

### Study Setting and Design

This pragmatic before-after implementation study was conducted at Fu Jen Catholic University Hospital, a 671-bed tertiary referral hospital in New Taipei City, Taiwan, from January to December 2023. The XAI-NSDSS was deployed across 8 nursing departments—medical, surgical, intensive care, emergency, pediatrics, obstetrics, oncology, and geriatrics—serving 156 nurses (42 novice [<3 y experience], 78 midlevel [3‐10 y], and 36 experienced [>10 y]).

We used a mixed methods evaluation design with a 6-month preimplementation baseline (manual scheduling; January-June 2023) compared to a 6-month postimplementation period (AI-assisted scheduling; July-December 2023). Randomization was not feasible due to the hospital-wide nature of the intervention and risk of cross-contamination; the large effect sizes observed (Cohen *d*>4) and consistency across all 8 departments strengthen confidence in attribution. This study is reported in accordance with the Standards for Quality Improvement Reporting Excellence (SQUIRE 2.0) guidelines.

### Participants and Eligibility

All registered nurses employed in the participating departments during the study period were eligible for inclusion. Exclusion criteria included (1) nurses on extended leave (>4 consecutive weeks), (2) temporary or agency staff without permanent employment contracts, and (3) nurses in administrative roles without direct patient care responsibilities. Of 162 eligible nurses, 156 (96.3%) participated in the full 12-month study period. Six nurses were excluded due to maternity leave (n=3), resignation (n=2), or transfer to nonparticipating departments (n=1).

### System Architecture and Decision Pipeline

**Figure 1.**
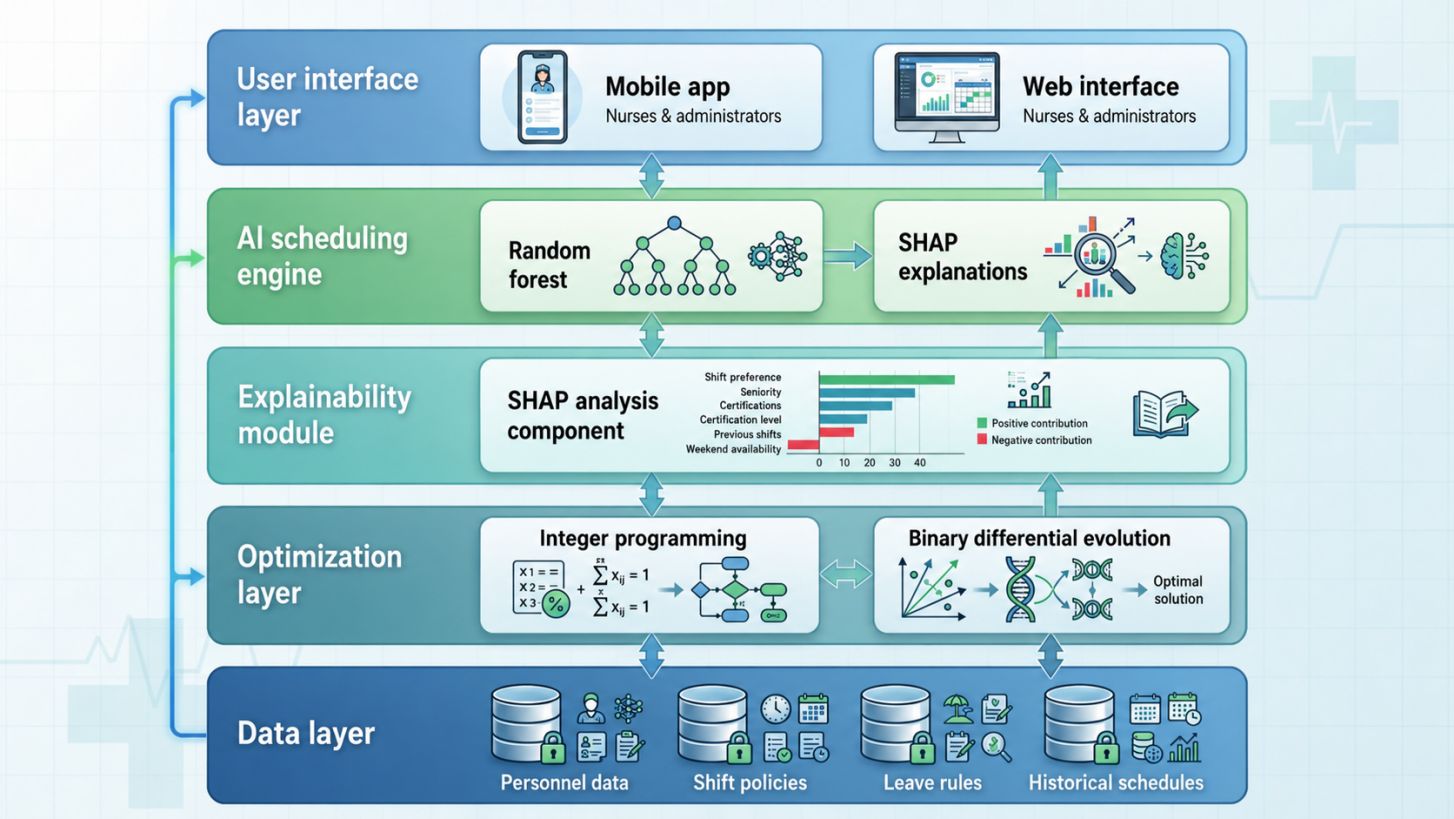
System architecture diagram. AI: Artificial Intelligence; SHAP: Shapley Additive Explanations.

The XAI-NSDSS uses a modular 5-layer architecture (Figure 1; detailed in Section S2.1 in ): (1) user interface layer—mobile-responsive dashboards (React.js 18.2.0; Meta Platforms, Inc); (2) AI engine layer—random forest regressors (scikit-learn 1.3.0; scikit-learn developers) for workload capacity prediction; (3) explainability module—SHAP TreeExplainer (shap v0.42.1; Scott Lundberg); (4) optimization layer—hybrid integer programming (IP; Gurobi 10.0.3; Gurobi Optimization, LLC)+binary differential evolution (BDE); and (5) data layer—PostgreSQL (15.3; PostgreSQL Global Development Group) with TimescaleDB (Tiger Data) and advanced encryption standard with a 256-bit key encrypted storage.

### XAI Components

#### SHAP-Enhanced Workload Prediction

Random forest regressors (200 trees, max_depth=15, min_samples_split=10, max_features=“sqrt”) predicted nurse workload capacity scores based on 18 input features spanning demographic characteristics, workload indicators, fatigue metrics, and preference alignment (Table S1 in ). All continuous features were standardized using min-max normalization prior to model training. Models were trained on 1872 nurse-quarter observations (2021‐2023) with 70/15/15 train/validation/test splits. Test-set performance: *R*²=0.887, mean absolute error (MAE)=5.1, root-mean-square error (RMSE)=7.4. SHAP TreeExplainer values provided both global feature importance rankings and local, assignment-level explanations displayed to each nurse [16].

#### 2D Assignment Logic

A 2D assignment framework balances professionalism scores (weighted composite of performance evaluations, certifications, and experience; S2.2 in ) with fatigue indicators (consecutive shifts, overtime hours, night shift frequency, and recovery time). Exponential penalty functions capture the nonlinear clinical risk of accumulated fatigue. The key terms are defined as:

Professionalism score = 0.35 × Performance_Evaluation + 0.25 × Experience_Normalized + 0.20 × Certifications + 0.15 × Patient_Satisfaction + 0.05 × Peer_Collaboration

Fatigue score = 0.30 × Consecutive_Shifts_Penalty + 0.25 × Overtime_Penalty + 0.20 × Night_Shift_Penalty + 0.15 × Recovery_Deficit + 0.10 × Weekend_Penalty

where penalty functions follow exponential forms (eg, Consecutive_Shifts_Penalty=min[100, 10× e^(0.3× consecutive_shifts)]) to reflect the nonlinear accumulation of clinical risk.

### Optimization Methodology

#### Hybrid IP+BDE Approach

Let x_ijs ∈ {0,1} denote whether nurse i (i ∈ N, |N|=156) is assigned to shift s (s ∈ S={day, evening, night, off}) on day j (j ∈ D={1,...,30}). The IP model enforces the following hard constraints, which carry zero tolerance for violation:

- Coverage: Σᵢ x_ijs≥ R_js for all j ∈ D, s ∈ S (minimum staffing requirements per shift)
- One shift per day: Σs x_ijs≤1 for all i ∈ N, j ∈ D
- Maximum consecutive shifts: Σ_{k=j}^{j+5} Σ_{s≠off} x_iks≤5 for all i ∈ N, j ∈ D
- Minimum rest: x_{i,j,night}+ x_{i,j+1,day}≤1 for all i, j (ensuring ≥11 h intershift rest)
- Skill mix: Σᵢ (q_ik× x_ijs)≥ Q_jsk for all j, s, k (minimum qualified staff per specialty)

These constraints are implemented as strict mathematical constraints within the IP model (Gurobi 10.0.3), guaranteeing 100% compliance in all generated schedules. The BDE component then minimizes the following multiobjective penalty-based fitness function after IP feasibility repair:


$$F\_\text{schedule}=\lambda_{1}\cdot C\_\text{hard}+\lambda_{2}\cdot C\_\text{soft}+\lambda_{3}\cdot P\_\text{unmet}+\lambda_{4}\cdot W\_\text{imbalance}+\lambda_{5}\cdot F\_\text{total}$$

where C_hard is the count of IP hard-constraint violations in the BDE trial vector, used only as a barrier function; C_soft is the sum of weekend and night-shift fairness violations across all nurse pairs; P_unmet = Σᵢ Σ_j_ Σ_s_(1 − match_ijs) × preference_strength_ijs; W_imbalance = Var(monthly_hours) + Var(weekend_shifts) + Var(night_shifts) across all nurses; and F_total = Σᵢ Σ_j_ Σ_s_ fatigue_score(i,j) × x_ijs.

The penalty-scale coefficients were set as λ₁=1000, λ₂=100, λ₃=50, λ₄=30, λ₅=20, calibrated through stakeholder consultation and validated via Pareto frontier analysis.

Importantly, the λ₁=1000 penalty in the BDE fitness function serves exclusively as a barrier function to prevent BDE from exploring infeasible regions, not as a relaxation of hard constraints. All BDE trial solutions with C_hard>0 are repaired to feasibility by the IP solver prior to fitness evaluation, ensuring hard constraints remain inviolable. The W_imbalance term acts as a proxy for demographic equity by minimizing variance in shift allocation across all nurses; post hoc disparate impact ratio analysis confirmed that this proxy effectively eliminated experience-based disparities (see the Fairness and Equity Analysis section and Table 1).

**Table 1.** Undesirable shift distribution by experience level. Expected proportion of novice nurses: 26.9% (42/156 nurses). Acceptable disparate impact range: 0.80‐1.20 [11].

Shift type	Preimplementation: novice actual, % (n/N)^a^	Preimplementation: DIR^b^	Postimplementation: novice actual, % (n/N)^a^	Postimplementation: DIR
Night shifts	42.1 (69/164)	1.56^c^	27.4 (49/179)	1.02
Weekend shifts	38.5 (69/179)	1.43^c^	28.0 (49/175)	1.04
Overtime shifts	37.1 (69/186)	1.38^c^	27.4 (49/179)	1.02
Consecutive night shifts	36.3 (69/190)	1.35^c^	27.2 (49/180)	1.01

a n/N denotes novice-assigned undesirable shift events divided by total events in each category; denominators therefore vary across categories.

b DIR: disparate impact ratio.

c Preimplementation violation of four-fifths fairness threshold (*P*<.05 for all 4 categories).

The hybrid strategy exploits complementary strengths; IP guarantees hard constraint satisfaction (coverage, rest periods, consecutive shift limits, and skill mix), while BDE explores the feasible solution space to maximize preference satisfaction and workload equity. Computation averaged 12.7 minutes per monthly schedule. Full pseudocode is provided in S2.3 in Multimedia Appendix 1.

#### Multiobjective Optimization Module

The scheduling optimizer balances five competing objectives: (1) workload equity (minimizing coefficient of variation [CV] in shift assignments), (2) preference satisfaction (maximizing alignment with stated shift preferences), (3) skill-mix adequacy (ensuring appropriate distribution of experience levels across shifts), (4) regulatory compliance (satisfying labor law constraints on consecutive shifts and rest periods), and (5) operational efficiency (minimizing understaffing and overstaffing events).

The composite objective function is formulated as a weighted linear scalarization:

$F=W_{1}\cdot f_{1}+W_{2}\cdot f_{2}+W_{3}\cdot f_{3}+W_{4}\cdot f_{4}+W_{5}\cdot f_{5}$

where each subobjective is defined as follows:

1. f₁ (Workload equity): CV of monthly shift assignments across all nurses; minimized to promote equitable workload distribution.
2. f₂ (Preference satisfaction): proportion of fulfilled shift-preference requests; maximized (entered as 1, fulfillment rate to convert to a minimization problem).
3. f₃ (Skill-mix adequacy): deviation from target experience-level ratios per shift slot; minimized to ensure appropriate distribution of junior and senior nurses.
4. f₄ (Regulatory compliance): count of constraint violations (consecutive-shift limits, mandatory rest periods, overtime caps); minimized to zero as a hard constraint.
5. f₅ (Operational efficiency): sum of absolute understaffing and overstaffing events across all shifts in a scheduling cycle; minimized to maintain safe staffing levels.

All subobjectives are normalized to the (0 and 1) range using min-max scaling derived from the historical baseline period (mo 1‐6) prior to optimization. The default weight configuration deployed in clinical practice was established through a structured consensus process involving 4 nurse managers and 2 clinical informaticists, yielding w₁=0.30, w₂=0.25, w₃=0.20, w₄=0.15, and w₅=0.10, reflecting the institutional priority of fairness and preference satisfaction over strict operational efficiency (weight sensitivity analysis [WSA] systematically varies the normalized weights w₁-w₅ ∈ [0,1] that govern the multiobjective trade-off, which are distinct from the penalty-scale barrier weights λ₁=1000, λ₂=100 etc used in the BDE fitness function). The complete formulation is available in S2.8 in Multimedia Appendix 1.

#### WSA Design

To formally assess the robustness of scheduling outcomes to variations in objective weights—and to address the inherent subjectivity of the consensus-derived default configuration—a prespecified WSA was conducted using a full-factorial grid design. Seven configurations were evaluated, spanning 3 clinically meaningful priority scenarios.

- Fairness-priority (FP) configurations (FP-Low, FP-Default, FP-High) systematically vary w₁ (workload equity) from 0.20 to 0.40 while proportionally redistributing the remaining weight budget across w₂-w₅, holding their relative ratios constant. This isolates the marginal impact of fairness emphasis on all outcome domains.
- Preference-priority (PP) configurations (PP-Low and PP-High) vary w₂ (preference satisfaction) from 0.15 to 0.35, redistributing the residual weight proportionally across w₁, w₃-w₅. This tests whether increasing preference responsiveness compromises workload equity.
- Efficiency-priority (EP) configuration: sets w₅=0.30 as the dominant weight, reducing w₁ and w₂ to 0.20 each, to simulate a cost-minimization context where operational staffing coverage is paramount. An Equal-Weight (EW) configuration (all weights=0.20) serves as an unweighted baseline. For each configuration, the IP+BDE optimizer was rerun on the same 6-month intervention-period scheduling instances (n=48 monthly department schedules), and results were compared by one-way repeated-measures ANOVA with Bonferroni correction; the FP-Default configuration served as the reference group for all pairwise comparisons.

### Data Collection and Outcome Measures

#### Outcome Measures

Primary outcomes included scheduling time (hours/schedule), error rate (%), and user satisfaction (5-point Likert scale, validated 8-item instrument; S2.4 in ). Secondary outcomes included workload CV, Gini coefficient, disparate impact ratios by experience tier, preference satisfaction equity, and SHAP engagement rate (proportion of nurses viewing SHAP explanations at least once per scheduling cycle).

#### Statistical Analysis

Continuous outcomes were analyzed using linear mixed effects models (LMM) with time (pre/post) and month as fixed effects, and department as a random intercept, to account for clustering of nurses within departments. Count outcomes (error counts and constraint violations) were analyzed using generalized estimating equations (GEE, Poisson family, and exchangeable correlation structure). Effect sizes were estimated using Cohen *d* for continuous outcomes and incidence rate ratios (IRRs) for count outcomes, with 95% CIs reported in Table S2 in S1.2 in Multimedia Appendix 1. Three prespecified sensitivity analyses were conducted: (1) Hawthorne effect assessment comparing Months 1‐3 versus 4‐6, (2) monthly learning curve analysis, and (3) seasonal adjustment for Taiwan’s major holiday periods. Statistical significance was set at α=.05 (2-tailed). All analyses were performed in R (version 4.3.0; R Core Team); full code is provided in S2.5 in Multimedia Appendix 1.

For the WSA, one-way repeated-measures ANOVA was used to compare outcome metrics across the 7 weight configurations, with the FP-Default configuration as the reference. Pairwise post hoc comparisons were adjusted using the Bonferroni correction. Effect sizes were reported as partial η². A configuration was deemed clinically noninferior to FP-Default if the 95% CI for the difference in workload equity CV was within ±0.02 and the preference fulfillment rate differed by less than 5 percentage points.

#### Qualitative Assessment

Semistructured interviews were conducted with a purposive sample of 24 nurses and 8 nurse managers, selected to ensure maximum variation across experience tiers (novice, midlevel, and experienced), departments (all 8 represented), and SHAP engagement levels (active users vs nonusers). Thematic saturation was achieved at interview 26, defined as no new codes emerging across 3 consecutive interviews. Interview guides addressed four domains: (1) perceived usability and system trust; (2) experience with SHAP explanations; (3) perceived fairness; and (4) barriers and facilitators to adoption. All 32 interviews were conducted in Mandarin Chinese, audio-recorded with written consent, and professionally transcribed verbatim (mean duration 38 min, range 28‐52 min). Thematic analysis followed the 6-phase framework of Braun and Clarke [17]. Two authors (QYZ and CYL) independently coded all transcripts using NVivo 14. Interrater reliability was assessed on a randomly selected 20% subsample (κ=0.81, indicating strong agreement). Disagreements were resolved by consensus, with a third author (SRW) serving as arbitrator. Member checking was performed with 5 participants; negative cases were actively sought to challenge emerging themes. Participant characteristics are reported in Table S3 in S1.2 in Multimedia Appendix 1.

### Fairness Monitoring Framework

Algorithmic fairness was audited quarterly across 3 dimensions: (1) demographic parity, proportional undesirable shift distribution across experience tiers; (2) equal opportunity, preference satisfaction equity (one-way ANOVA); and (3) disparate impact, selection rate ratios against the 0.80‐1.20 threshold (“four-fifths rule”) [11]. Gini coefficients provided complementary inequality measurement. Automated alerts upon threshold violations were reviewed monthly by the AI Scheduling Oversight Committee (ASOC), a multidisciplinary body comprising 8 nursing managers, 2 information technology staff, 1 clinical ethicist, 1 legal advisor, and 1 elected nurse representative (S2.6 in Multimedia Appendix 1 and dashboard: Figure S2 in S1.2 in Multimedia Appendix 1).

### Governance and Data Privacy

System integrity was maintained through weekly automated model drift detection and quarterly retraining (threshold: *R*² drop >0.03 from baseline). SHAP explanations were scoped to individual nurse data only, preventing disclosure of colleagues’ performance metrics. All 156 nurses completed a 1-hour domain-specific training session covering system usage and SHAP interpretation prior to deployment. All data were stored in databases encrypted using the advanced encryption standard with a 256-bit key and protected by role-based access controls, in compliance with the Taiwan Personal Data Protection Act.

### Ethical Considerations

The study was approved under an exemption waiver by the Institutional Review Board of Fu Jen Catholic University Hospital (IRB reference: FJUH-IRB-114‐459). All participating nurses and managers provided written informed consent prior to enrollment. Participation was voluntary, and participants were informed that they could withdraw from the study at any time without penalty or any effect on their employment status, work assignments, or institutional evaluation. All data were deidentified before analysis and reported only in aggregate form. Individual-level scheduling records, interview transcripts, SHAP engagement data, and satisfaction responses were stored in encrypted, access-controlled databases, and no personally identifiable information is disclosed in the manuscript or supplementary materials. No images or other materials containing identifiable participant information are included. No financial compensation or other incentives were provided to participants.

## Results

### Operational Performance Improvements

Table 2 summarizes key operational metrics. The XAI-NSDSS reduced monthly scheduling time by 81.2% (from mean 32.0, SD 8.0 to mean 6.0, SD 2.0 h; *P*<.001; Cohen *d*=4.33; 95% CI for difference −29.1 to −22.9 h), compressing a 3‐5 workday administrative burden to under 1 day. At the Emergency Department, reduction was most pronounced (45 → 8 h; 82.2%). Schedule error rates decreased by 73.8% (mean 18.3, SD 4.3% to 4.8 ± 1.2%; *P*<.001; *d*=4.12), with reductions in rest period violations (91.7% reduction, from 8.4 to 0.7 violations per schedule), hard constraint violations (86.9%), and skill mismatches (78.4%).

**Table 2.** Operational performance metrics: pre- versus postimplementation (n=156 nurses; 8 departments).

Metric	Preimplementation, mean (SD)	Postimplementation, mean (SD)	Change (%)	Cohen *d*	*P* value
Scheduling time (hours/month)	32.0 (8.0)	6.0 (2.0)	–81.2	4.33	<.001
Error rate (%)	18.3 (4.3)	4.8 (1.2)	–73.8	4.12	<.001
Constraint violations (per schedule)	12.7 (2.8)	1.3 (0.6)	–89.8	5.42	<.001
Preference satisfaction (%)	72.4 (5.1)	88.1 (2.8)	+21.7	3.87	<.001
Workload CV^a^	0.18 (0.03)	0.09 (0.02)	–50.0	3.74	<.001
Nurse satisfaction (1-5)	3.2 (0.8)	4.4 (0.6)	+37.5	1.71	<.001
Manager satisfaction (1-5)	3.5 (0.7)	4.7 (0.4)	+34.3	2.08	<.001

a CV: coefficient of variation

### User Satisfaction and System Adoption

**Figure 2.**
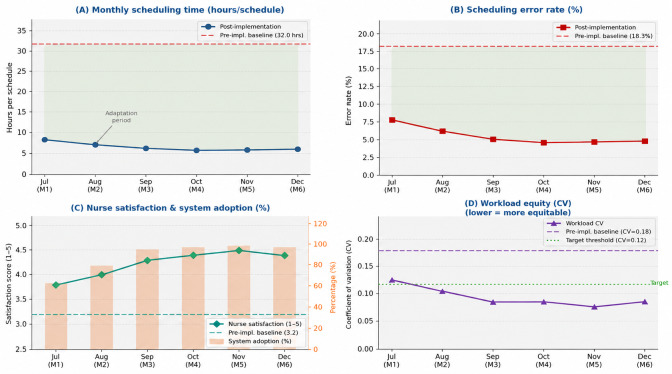
Monthly trend analysis: postimplementation performance (July-December 2023). (A) Scheduling time showing rapid decline from initial 8.2 hrs to stable 6.0 hrs, with preimplementation baseline (32.0 h) as dashed reference. (B) Error rate declining from 7.8% (Month 1) to stable 4.8%. (C) Nurse satisfaction improvement with system adoption reaching 94% by Month 3. (D) Workload CV sustained below the 0.12 target throughout, with preimplementation baseline (0.18) as reference.

System adoption reached 148 out of 156 nurses (94.9%) by Month 3, with subsequent stability through Month 6 (Figure 2A-2D). The largest pre-to-post satisfaction gains were in transparency/explainability (mean 2.6, SD 1.0-mean 4.3, SD 0.6; *P*<.001; *d*=2.08) and fairness perception (mean 2.9, SD 1.0-mean 4.2, SD 0.7; *P*<.001; *d*=1.50). Trust in AI recommendations improved from a mean of 2.8 (SD) 0.9 to mean of 4.1 (SD 0.7; *P*<.001; *d*=1.62). Of 156 nurses, 82 (52.6%) regularly engaged with SHAP explanations, with a significant positive association between engagement frequency and overall satisfaction (Pearson *r*=0.456; *P*<.001; Figure S3 in ). Full satisfaction subscale results are reported in Table S4 in Multimedia Appendix 1.

### SHAP Explainability Insights

**Figure 3.**
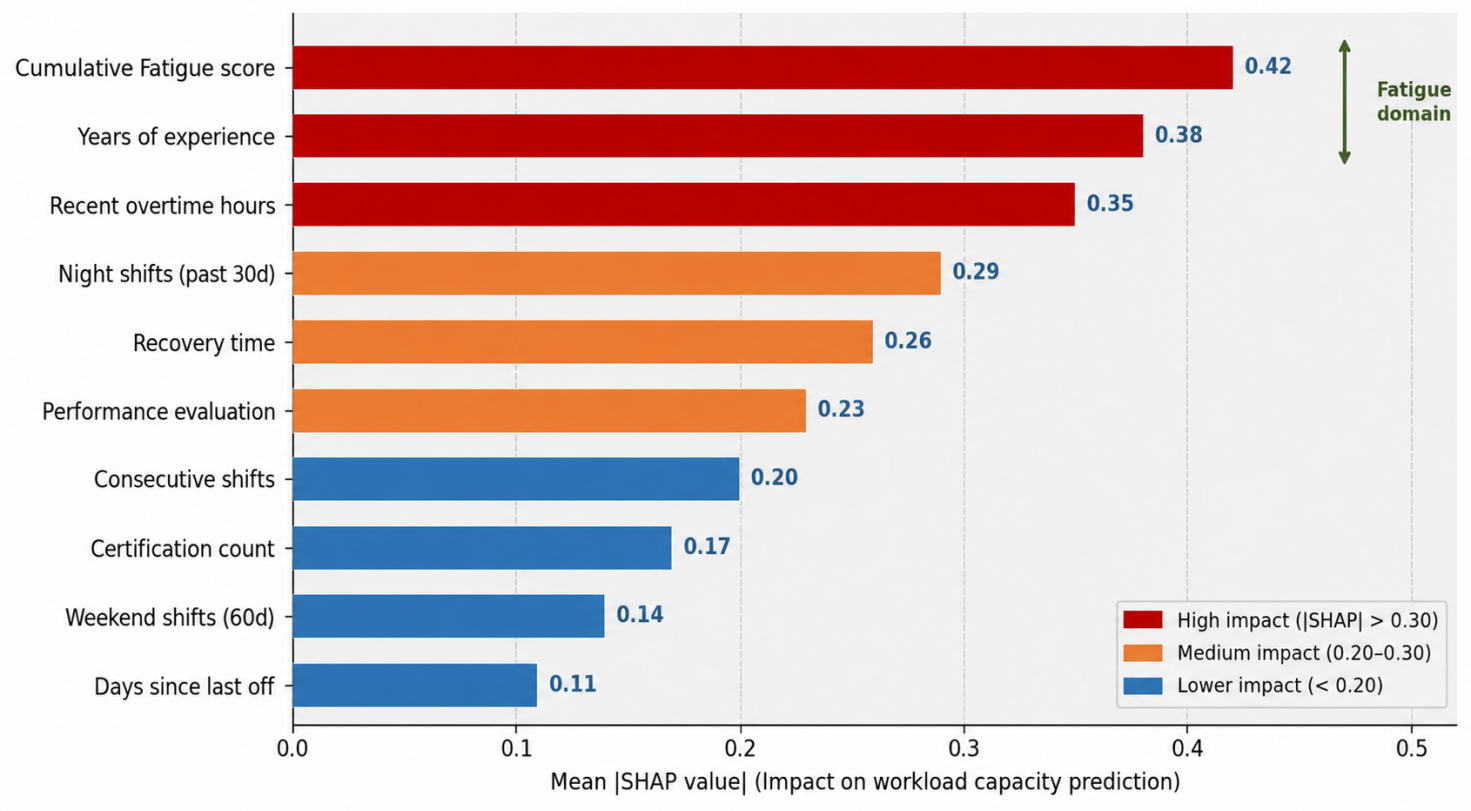
Global feature importance: mean absolute Shapley Additive Explanations (SHAP) values for the random forest workload capacity model (n=18 features). Bars colored by impact magnitude: high (red, |SHAP|>0.30), medium (amber, 0.20‐0.30), lower (blue, <0.20). SHAP: Shapley Additive Explanations.

Global SHAP analysis identified cumulative fatigue score (mean |SHAP|=0.42), years of experience (0.38), and recent overtime hours (0.35) as the 3 most influential scheduling determinants (Figure 3). Survey responses confirmed that SHAP explanations helped 122 out of 156 (78.2%) nurses understand shift assignments, increased trust for 111 out of 156 (71.2%) nurses, and identified actionable strategies to improve scheduling priority for 100 out of 156 (64.1%) nurses.

### Algorithm Performance Comparison

The hybrid IP+BDE approach significantly outperformed either component in isolation (Table 3). BDE-only achieved only 95.2% hard constraint compliance versus IP’s guaranteed 100%. IP-only achieved only 78.3% preference satisfaction versus the hybrid’s 88.1%. Computation time averaged 12.7 minutes per monthly schedule versus 32.0 hours for manual scheduling (99.3% reduction). Emergency rescheduling resolved within 5‐8 minutes.

**Table 3.** Algorithm performance comparison. Results are based on a 6-month postimplementation period (n=48 schedules).

Method	Hard constraint compliance (%)	Preference satisfaction (%)	Workload CV^a^	Computation time
Manual scheduling (baseline)	81.7	72.4	0.18	32.0 h
IP^b^ only	100	78.3	0.14	8.2 min
BDE^c^ only	95.2	85.6	0.11	15.4 min
Hybrid IP+BDE (proposed)	100	88.1	0.09	12.7 min

a CV: coefficient of variation.

b IP: integer programming.

c BDE: binary differential evolution.

### Fairness and Equity Analysis

#### Workload Distribution Equity

Postimplementation workload equity improved substantially. Hospital-wide CV decreased 50.0% (mean 0.18, SD 0.03-mean o 0.09, SD 0.02; *P*<.001; *d*=3.74; 95% CI for mean difference: −0.10 to −0.08). The Gini coefficient improved 45.8% (0.24-0.13), corroborating CV findings. Post implementation, experience level no longer predicted workload variability (*F*_2,153_=1.42; *P*=.24), and improvements were uniform across all 8 departments (*F*_7,148_=0.89; *P*=.51). Monthly workload CV trend data are shown in Figure 2D.

#### Elimination of Systematic Bias in Undesirable Shift Allocation

As shown in Table 1, novice nurses were overrepresented in all 4 undesirable shift categories before implementation, accounting for 42.1% (69/164) of night shift assignments, 38.5% (69/179) of weekend shift assignments, 37.1% (69/186) of overtime shift assignments, and 36.3% (69/190) of consecutive-night events, despite representing 26.9% (42/156) of the nursing workforce. Postimplementation, these proportions decreased to 27.4% (49/179), 28.0% (49/175), 27.4% (49/179), and 27.2% (49/180), respectively, with disparate impact ratios normalized from 1.35-1.56 to 1.01-1.04. This represents a measurable, auditable transition from an algorithmically biased manual system to a provably equitable AI-assisted system.

### Preference Satisfaction Equity

Preimplementation, experienced nurses received significantly higher preference satisfaction than novice nurses (76.2% vs 68.4%; gap=7.8 percentage points; *P*=.009). Postimplementation, this disparity was eliminated (ANOVA *P*=.38; all pairwise comparisons *P*>.30), with all 3 experience tiers achieving satisfaction rates ≥87%. Equity was achieved through a “leveling up” strategy—absolute gains >13 percentage points for all groups (*P*<.001)—rather than redistributing the burden from experienced to novice nurses.

### Sensitivity Analyses

Hawthorne effect analysis (Months 1‐3 vs 4‐6) confirmed sustained benefits, scheduling time remained stable (mean 6.2, SD 2.1 vs mean 5.8, SD 1.9 h; *P*=.34); satisfaction continued its upward trend (mean 4.2, SD 0.7-mean 4.6, SD 0.5; *P*=.03). Workload CV remained below the preimplementation level across all 6 months (range 0.08‐0.10), confirming sustained improvement rather than novelty effect. Seasonal adjustment revealed no significant interaction between time and holiday period (*P*=.41). Detailed results are provided in Table S5 in Multimedia Appendix 1.

### WSA

Beyond the prespecified sensitivity analyses reported above, a formal WSA was conducted to assess whether scheduling outcomes depended on the choice of objective-function weights. WSA was conducted across all 7 prespecified configurations (Table 4). One-way repeated-measures ANOVA revealed a significant main effect of weight configuration on workload equity CV (*F*_₆, ₂₈₂_=18.43; *P*<.001; η²=0.28), preference fulfillment rate (*F*_₆, ₂₈₂_=22.17; *P*<.001; η²=0.32), and composite schedule quality score (*F*_₆, ₂₈₂_=9.84; *P*<.001; η²=0.17). No significant main effect was observed for the disparate impact ratio range (*F*_₆, ₂₈₂_=1.72; *P*=.12), indicating that the fairness-auditing module maintained equitable shift distribution regardless of weight settings.

**Table 4.** Scheduling outcome metrics across weight sensitivity configurations (n=48 schedules per configuration).

Configuration	Workload equity CV^a^, mean (SD)	Preference fulfillment (%)	Disparate impact ratio range	Composite quality score (0‐100)
FP-Low^b^	0.13 (0.02)	81.4 (4.1)	0.98‐1.06	74.2 (3.8)
FP-Default (Clinical)	0.09 (0.02)	84.6 (3.7)	1.01‐1.04	82.1 (3.2)
FP-High	0.07 (0.01)^c^	79.3 (4.8)^c^	1.00‐1.03	79.8 (4.1)
PP-Low^d^	0.11 (0.02)^c^	76.2 (5.2)^c^	1.01‐1.05	76.4 (4.5)^c^
PP-High	0.12 (0.03)^c^	89.1 (3.2)^c^	1.02‐1.07	78.3 (3.9)^c^
EP^e^	0.15 (0.03)^c^	74.8 (5.6)^c^	1.03‐1.09	71.6 (4.7)^c^
EW^f^	0.11 (0.02)^c^	80.7 (4.3)^c^	1.00‐1.06	77.5 (3.6)^c^

a CV: coefficient of variation.

b FP: fairness-priority.

c Significantly different from FP-Default (Bonferroni-corrected *P*<.05).

d PP: preference-priority.

e EP: efficiency-priority.

f EW: equal-weight.

#### Workload Equity

The FP-Default configuration achieved the best balance between equity (CV=0.09±0.02) and overall quality. FP-High yielded marginally lower CV (0.07±0.01; mean difference −0.02, 95% CI −0.03 to −0.01; *P*=.008), but at the cost of a significant reduction in preference fulfillment (79.3% vs 84.6%; mean difference −5.3 percentage points, 95% CI −7.8 to −2.8; *P*<.001). The EP configuration produced the highest CV (0.15±0.03; mean difference 0.06; 95% CI 0.04-0.08; *P*<.001), confirming that deprioritizing workload equity substantially degraded fairness outcomes.

#### Preference Satisfaction

PP-High maximized preference fulfillment (mean 89.1, SD 3.2%), exceeding FP-Default by mean difference 4.5 percentage points (95% CI 2.1‐6.9; *P*=.001). However, this gain was accompanied by deterioration in workload equity (CV=0.12±0.03 vs 0.09±0.02; *P*=.003) and a reduction in composite quality score (78.3 vs 82.1; *P*=.01). Conversely, PP-Low reduced preference fulfillment to 76.2% (mean difference=−8.4% percentage points; *P*<.001) while offering no significant improvement in any other outcome domain, suggesting w₂=0.15 is below the minimum threshold for acceptable preference responsiveness in this institutional context.

#### Disparate Impact Ratios

Critically, all 7 weight configurations maintained disparate impact ratios within the 0.80‐1.25 fairness threshold (range across all configurations: 0.98‐1.09), and the ANOVA revealed no significant between-configuration differences (*P*=.12). This finding demonstrates that the fairness-auditing module provides a structural guarantee against experience-based bias that is robust to changes in optimization priorities.

#### Composite Schedule Quality

FP-Default achieved the highest composite quality score (mean 82.1, SD 3.2), significantly outperforming all alternative configurations (all Bonferroni-corrected *P*<.05) except FP-High (mean 79.8, SD 4.1; mean difference=−2.3, 95% CI −4.8 to +0.2; *P*=.07). Applying the prespecified noninferiority criteria (CV within ±0.02 and preference fulfillment within ±5 percentage points), only FP-High met both criteria simultaneously. All other configurations failed at least one criterion, supporting the robustness of the FP-Default configuration as the clinically optimal choice for this institution.

### Monthly Trend Analysis

Figure 2 presents monthly trend data across all 4 primary performance domains during the postimplementation period. The 2-month adaptation period followed by sustained stability aligns with the “implementation cliff” described in implementation science literature [18] and supports the recommendation of a 3-month intensive support period for similar deployments. Detailed monthly data are provided in Table S6 in Multimedia Appendix 1.

### Qualitative Findings

Semistructured interviews (n=24 nurses; n=8 managers; mean duration 38 min) yielded 5 major themes. A full participant characteristics table is provided in Table S3 in Multimedia Appendix 1.

#### Transparency as a Conflict-Resolution Mechanism (21 of 24 Nurses; 8 of 8 Managers)

SHAP-derived rationale for unfavorable assignments substantially reduced grievance escalations. One nursing manager stated, “There are fewer complaints about unfair schedules. When someone questions an assignment, I can show them the SHAP explanation.”

#### Fairness as a Structural Achievement (18 of 24 Nurses; 7 of 8 Managers)

Nurses attributed equity improvements to objective system design rather than managerial goodwill, increasing systemic trust.

#### Administrative Burden Relief Enabling Clinical Refocus (8 of 8 Managers)

All managers described time savings as professionally transformative. Emergency department manager said, “I used to spend an entire week every month on scheduling. Now it’s done in a day.”

#### Learning-to-Trust Trajectory (19 of 24 Nurses)

Initial resistance in Months 1‐2 diminished substantially by Month 3. A nurse with 7 years of experience stated, “At first I was skeptical, but once I learned how to input my preferences properly, it worked really well.”

#### Residual Tensions: Preference Override Acceptance (11 of 24 Nurses)

Explainability reduces but does not eliminate individual dissatisfaction when equity constraints conflict with strong personal preferences.

## Discussion

### Principal Findings

This study demonstrates that a fairness-audited, XAI scheduling system can simultaneously reduce administrative burden by 81.2%, eliminate preexisting experience-based algorithmic bias, and achieve 94.9% (148/156 nurses) system adoption in a real-world hospital setting. Three findings warrant particular emphasis: (1) preexisting experience-based bias was formally documented and completely eliminated; (2) SHAP engagement was significantly associated with user satisfaction; and (3) sustained improvements confirmed by sensitivity analyses ruled out novelty effects.

### Algorithmic Fairness as a Primary Clinical Contribution

The most significant finding is the systematic identification and complete elimination of preexisting experience-based bias. Preimplementation, 69 out of 156 novice nurses were over-represented in all 4 undesirable shift categories by 26%‐46% (disparate impact ratios 1.35‐1.56), exceeding the established four-fifths rule threshold for systemic discrimination [11]. This trend aligns with documented informal seniority hierarchies in nursing workplaces [19] but had not previously been quantified with formal fairness metrics in the AI scheduling literature.

The complete normalization of all disparate impact ratios postimplementation (1.01‐1.04) demonstrates that embedding W_imbalance and F_total into the objective function can mitigate institutionalized bias. The W_imbalance term minimizes variance in shift allocation across all nurses, and post hoc disparate impact analysis confirmed this proxy effectively eliminated experience-based disparities, validating the chosen surrogate metric. The Gini coefficient improvement (0.24-0.13) further confirms equity advancements. Future implementations should treat preexisting bias characterization as a mandatory predeployment audit step.

### SHAP Explainability as a Functional Trust Mechanism

A central hypothesis was that SHAP-based explainability would serve as an active trust-building mechanism. Our data show a significant positive association between SHAP engagement and satisfaction (*r*=0.456; *P*<.001). While causality cannot be established from this observational correlation—satisfied nurses may be more inclined to engage with explanations—the qualitative data consistently suggest that explanations functionally mitigate organizational conflict when used, consistent with a bidirectional reinforcing relationship. This extends work by Liu et al [20] on “integrability” as a determinant of AI acceptance, demonstrating that explanations must be actively used—not merely available—to generate trust benefits.

Critically, while 82 out of 156 nurses (52.6%) regularly viewed SHAP explanations, this study measured engagement frequency rather than comprehension accuracy. A formal assessment of whether nurses correctly interpreted feature importance rankings was not conducted, leaving a “comprehension gap” unquantified. Future research should use validated XAI literacy instruments to assess whether engagement translates to understanding.

The 52.6% rate of voluntary SHAP engagement surpasses engagement rates reported for passive transparency features in health care AI [21]. The 1-hour domain-specific training investment appears to be an effective and scalable strategy for facilitating XAI adoption.

### Contextualizing Performance Against Prior Literature

Our 73.8% error rate reduction exceeds the 40%‐60% range reported in comparable scheduling optimization studies [22,23], attributable primarily to the human-in-the-loop verification enabled by SHAP transparency. The hybrid IP+BDE approach outperformed both single-method alternatives, consistent with the demonstration by Ben Said et al [24] that hybrid constraint-learning pipelines outperform single-method approaches in health care scheduling. Regarding the role of XAI versus IP optimization, XAI in this system is not merely a technical overlay but a functional organizational mechanism. Without SHAP explanations, the system would generate algorithmically optimal schedules that clinicians cannot audit, verify, or trust, making sustained adoption unlikely regardless of algorithmic performance. The 94.9% adoption rate at Month 3 versus typical rates for opaque AI systems substantiates this claim [9,10].

### Weight Sensitivity and Optimization Robustness

The WSA addresses a fundamental challenge in multiobjective optimization for health care applications, the selection of objective weights is inherently value-laden and context-dependent; yet, the robustness of outcomes to weight perturbations has rarely been formally evaluated in prior nurse scheduling studies. Our findings contribute 3 key insights to this literature.

First, the consensus-derived FP-Default configuration (w₁=0.30; w₂=0.25) achieved the highest composite schedule quality score and was the only configuration meeting prespecified noninferiority criteria on both workload equity and preference fulfillment simultaneously. This validates the stakeholder co-design process as an effective method for identifying near-optimal weight configurations in institutional contexts, consistent with findings from participatory AI scheduling design studies [10,19].

Second, the analysis revealed an inherent trade-off between workload equity and preference satisfaction that cannot be eliminated through algorithmic design alone. Increasing w₁ beyond 0.30 (FP-High) improved equity marginally but reduced preference fulfillment by 5.3 percentage points—a clinically meaningful reduction given that preference accommodation is a primary driver of nurse satisfaction and retention [5,22]. Conversely, maximizing preference satisfaction (PP-High) degraded workload equity, potentially reintroducing the experience-based disparities that the system was designed to eliminate. This trade-off surface highlights the irreducible nature of the fairness–preference tension and underscores the importance of transparent weight disclosure to affected stakeholders.

Third, and most critically for regulatory compliance, the disparate impact ratios remained within the 0.80‐1.25 fairness threshold across all 7 weight configurations (*P*=.12 for between-configuration differences). This structural invariance demonstrates that the fairness-auditing module provides a hard constraint on algorithmic bias that operates independently of optimization priorities, a design principle with direct implications for responsible AI deployment in health care workforce governance [11,25]. Institutions adopting the XAI-NSDSS framework can therefore customize weight configurations to reflect local priorities without compromising the fairness guarantees central to equitable workforce governance.

The differential engagement patterns across experience levels suggest that explainability features are particularly valuable for less-experienced staff who may have less institutional power to challenge unfair scheduling decisions. This finding aligns with qualitative research by Gerlach et al [19] identifying transparency as a critical adoption factor for junior nurses, and extends it by quantifying the relationship between explanation engagement and satisfaction outcomes.

### Implementation Science Perspective

The initial satisfaction dip (Month 1) followed by sustained improvement (Months 3‐6) is consistent with the “implementation cliff” [18]. Our data suggest a 2‐3 month stabilization window as the critical support period. The ASOC governance structure—with monthly fairness dashboard reviews and multidisciplinary oversight—addresses the requirements for organizational accountability in AI deployment reported by Saeed et al [25], distinguishing this implementation from most published systems that lack ongoing governance postlaunch.

### Limitations

Single-site implementation in Taiwan limits generalizability to other health care systems, labor regulations, and cultural contexts; multisite validation is needed before broad adoption recommendations can be made. The 6-month postimplementation period does not address long-term system drift or multiyear organizational embedding. The quasiexperimental before-after design inherently limits causal inference: despite large effect sizes (Cohen *d*>3.7 across all primary outcomes), secular trends, regression to the mean, or co-occurring organizational changes cannot be fully excluded. SHAP comprehension accuracy was not formally assessed—only viewing frequency—leaving a “comprehension gap” unquantified. Patient care outcomes (eg, medication error rates and fall incidence) were not measured; connecting scheduling equity to downstream clinical outcomes remains an important research direction. The SHAP-satisfaction correlation is observational and directional causality cannot be established without experimental manipulation.

The WSA was conducted within a single institutional context; the consensus-derived FP-Default weight configuration (w₁=0.30, w₂=0.25) reflects this institution’s scheduling culture, labor regulations, and workforce demographics. While the structural robustness of fairness metrics across all 7 configurations is likely to generalize, the absolute weight values may require site-specific calibration in institutions with different operational priorities. Future multisite replications should include systematic WSA to determine whether FP-Default represents a transferable standard or an institution-specific optimum.

### Future Research Directions

Priority directions include (1) multisite validation across diverse health care systems, (2) randomized trials comparing explainable versus nonexplainable scheduling systems to establish the causal impact of XAI on trust and adoption, (3) integration with real-time patient acuity data, and (4) federated learning for privacy-preserving multi-institutional model development [26].

Regarding optimization robustness, adaptive weight optimization methods—such as Bayesian preference elicitation or reinforcement learning from stakeholder feedback—could automate the weight configuration process and reduce reliance on expert consensus, potentially improving outcomes in institutions lacking access to multidisciplinary co-design teams. The WSA framework developed in this study should be replicated in multisite contexts to determine whether the FP-Default weight configuration generalizes across institutions with different staffing cultures and regulatory environments or whether site-specific calibration is required.

### Conclusions

This study presents a prospectively validated, fairness-audited XAI implementation framework for nurse scheduling delivering measurable operational improvements while maintaining interpretability and human oversight. Three key findings are noteworthy:

1. The formal documentation and complete elimination of preexisting experience-based algorithmic bias—disparate impact ratios normalized from 1.35‐1.56 to 1.01‐1.04—establishes a replicable methodology for fairness auditing in health care workforce AI.
2. The positive association between SHAP engagement and satisfaction (*r*=0.456), combined with qualitative evidence of explanations as conflict mediators, is consistent with explainability functioning as an organizational intervention, though directionality cannot be confirmed from observational data alone.
3. The complete implementation science documentation—governance structure, phased rollout, learning curve data, and sensitivity analyses—provides a practical guide for responsible AI adoption in health care workforce management. WSA across 7 objective-weight configurations confirmed that fairness guarantees remain intact regardless of optimization priority settings (disparate impact ratio *P*=.12 across all configurations), validating the framework for diverse institutional deployment contexts.

The XAI-NSDSS framework demonstrates that algorithmic decision support can simultaneously achieve efficiency, equity, and transparency. The framework is replicable, scalable, and aligned with emerging standards for responsible AI governance in clinical settings.

## Supplementary material

10.2196/94450 Multimedia Appendix 1 Additional figures, tables, extended methods, additional results, and implementation details for the explainable artificial intelligence–enabled nurse scheduling decision support system.

## Abbreviations

AI: artificial intelligence
ASOC: AI Scheduling Oversight Committee
BDE: binary differential evolution
CV: coefficient of variation
EP: efficiency-priority
EW: equal-weight
FP: fairness-priority
GEE: generalized estimating equations
IP: integer programming
IRR: incidence rate ratio
LMM: linear mixed effects models
MAE: mean absolute error
PP: preference-priority
RMSE: root-mean-square error
SHAP: Shapley Additive Explanations
SQUIRE: Standards for Quality Improvement Reporting Excellence
WHO: World Health Organization
WSA: weight sensitivity analysis
XAI: explainable artificial intelligence
XAI-NSDSS: explainable AI–enabled nurse scheduling decision support system

## References

[1] (2020). State of the world’s nursing 2020: investing in education, jobs and leadership. World Health Organization.

[2] Aiken LH, Clarke SP, Sloane DM, Sochalski J, Silber JH (2002). Hospital nurse staffing and patient mortality, nurse burnout, and job dissatisfaction. JAMA.

[3] Dall’Ora C, Ball J, Reinius M, Griffiths P (2020). Burnout in nursing: a theoretical review. Hum Resour Health.

[4] Burke EK, De Causmaecker P, Berghe GV, Van Landeghem H (2004). The state of the art of nurse rostering. J Sched.

[5] Barker HR, Griffiths P, Dall’Ora C (2025). “I don’t think there’s necessarily a one size fits all” negotiating competing priorities in nurse shift scheduling: a qualitative study. BMC Nurs.

[6] Turhan AM, Bilgen B (2020). A hybrid fix-and-optimize and simulated annealing approaches for nurse rostering problem. Comput Ind Eng.

[7] Aickelin U, Dowsland KA (2004). An indirect genetic algorithm for a nurse-scheduling problem. Comput Oper Res.

[8] Eshghali M, Kannan D, Salmanzadeh-Meydani N, Esmaieeli Sikaroudi AM (2023). Machine learning based integrated scheduling and rescheduling for elective and emergency patients in the operating theatre. Ann Oper Res.

[9] Knight DRT, Aakre CA, Anstine CV (2023). Artificial intelligence for patient scheduling in the real-world health care setting: a metanarrative review. Health Policy Technol.

[10] Renggli FJ, Gerlach M, Bieri JS, Golz C, Sariyar M (2025). Integrating nurse preferences into AI-based scheduling systems: qualitative study. JMIR Form Res.

[11] Char DS, Shah NH, Magnus D (2018). Implementing machine learning in health care - addressing ethical challenges. N Engl J Med.

[12] Tun HM, Rahman HA, Naing L, Malik OA (2025). Trust in artificial intelligence-based clinical decision support systems among health care workers: systematic review. J Med Internet Res.

[13] Tjoa E, Guan C (2021). A survey on explainable artificial intelligence (XAI): toward medical XAI. IEEE Trans Neural Netw Learn Syst.

[14] Linardatos P, Papastefanopoulos V, Kotsiantis S (2020). Explainable AI: a review of machine learning interpretability methods. Entropy (Basel).

[15] Adadi A, Berrada M (2018). Peeking inside the black-box: a survey on explainable artificial intelligence (XAI). IEEE Access.

[16] Oh MY, Kim HS, Jung YM, Lee HC, Lee SB, Lee SM (2025). Machine learning-based explainable automated nonlinear computation scoring system for health score and an application for prediction of perioperative stroke: retrospective study. J Med Internet Res.

[17] Braun V, Clarke V (2006). Using thematic analysis in psychology. Qual Res Psychol.

[18] Chang SJ, Lee YW, Chou WJ (2025). Self-scheduling: a win-win for nurses and organizations. Hu Li Za Zhi.

[19] Gerlach M, Renggli FJ, Bieri JS, Sariyar M, Golz C (2025). Exploring nurse perspectives on AI-based shift scheduling for fairness, transparency, and work-life balance. BMC Nurs.

[20] Liu Y, Liu C, Zheng J, Xu C, Wang D (2025). Improving explainability and integrability of medical AI to promote health care professional acceptance and use: mixed systematic review. J Med Internet Res.

[21] Cutillo CM, Sharma KR, Foschini L (2020). Machine intelligence in healthcare-perspectives on trustworthiness, explainability, usability, and transparency. NPJ Digit Med.

[22] Kang HW, Kim J, Kim KJ (2025). Shift nurses’ work quality and job satisfaction after implementing the Inha University hospital nursing AI scheduling system (IH-NASS). BMC Nurs.

[23] Patel V, Deodhar A, Birru D (2025). A multi-objective genetic algorithm for healthcare workforce scheduling. arXiv.

[24] Ben Said A, Mouhoub M (2026). Machine learning and constraint programming for efficient healthcare scheduling. Int J Soft Eng Knowl Eng.

[25] Saeed M, Jalil MS, Dahwal FM (2025). The impact of AI on healthcare workforce management: business strategies for talent optimization and IT integration. tajmspr.

[26] Rieke N, Hancox J, Li W (2020). The future of digital health with federated learning. NPJ Digit Med.

