# Supplementary material for: Explainable AI for Equitable Nurse Scheduling: Pragmatic Pre-Post Implementation Study
Source: JMIR Nurs. 2026 Jul 2;9:e94450. doi: 10.2196/94450 (PMC13328950; doi:10.2196/94450)
Supplement: Multimedia Appendix 1 [file nursing-v9-e94450-s001.docx]

# Multimedia Appendix 1

# Explainable AI for Equitable Nurse Scheduling: A Pragmatic Pre-Post Implementation Study

*Hai-Pei Hsu, Ben-Chang Shia, Szu-Ming Peng, Chiu-Yang Chang, Chiung-Yun Lo, Sheng-Ru Wang*

*Corresponding Author: Sheng-Ru Wang (wang.clinic@gmail.com)*

*Last Updated: April 2nd, 2026*

# Table of Contents

## Appendix S1. Supplementary Figures and Tables

1.1 Supplementary Figures (S1–S3)

1.2 Supplementary Tables (S1–S7)

## Appendix S2. Extended Methods

2.1 Five-Layer System Architecture

2.2 Two-Dimensional Assignment Logic

2.3 Algorithm Pseudocode (IP + BDE)

2.4 Interview Protocols and Survey Instruments

2.5 Statistical Model Code (R)

2.6 Governance and Data Privacy Details

2.7 Random Forest Model Specifications

2.8 Multi-Objective Fitness Function (Complete Formulation)

## Appendix S3. Additional Results

3.1 Extended Fairness Analysis

3.2 Department-Level Breakdowns

3.3 Global Feature Importance of SHAP Values

3.4 Monthly Trend Data

# Appendix S4. Extended Discussion

4.1 Detailed Ethical Analysis

4.2 Implementation Guidelines

4.3 Technical Specifications for Replication

# Appendix S1. Supplementary Figures and Tables

## S1.1 Supplementary Figures


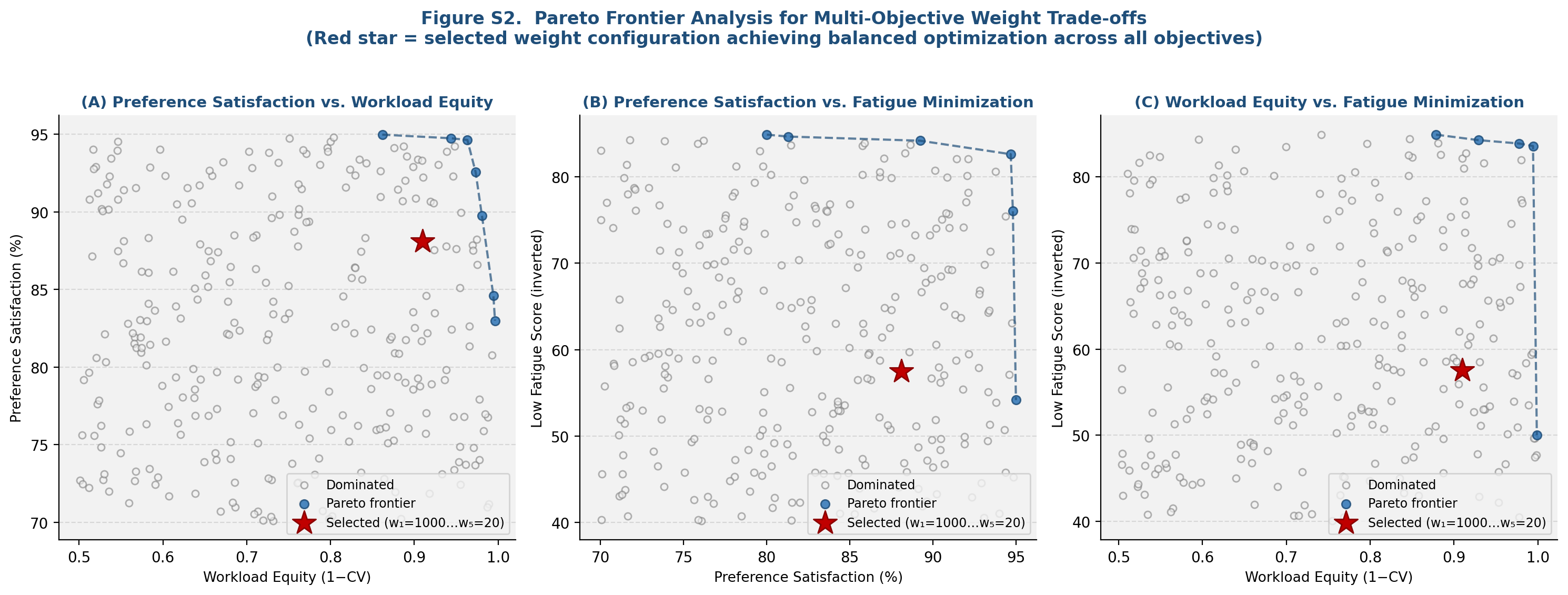


Figure S1. Pareto Frontier Analysis for Multi-Objective Weight Trade-offs. (A–C) Pareto frontier plots showing trade-offs between key objectives. Gray circles represent dominated (suboptimal) solutions; blue circles represent Pareto frontier solutions; dashed lines show fitted curves. Red stars indicate selected weight configuration (w₁=1000, w₂=100, w₃=50, w₄=30, w₅=20). (A) Preference satisfaction vs. workload equity: selected point achieves 88.1% satisfaction with CV=0.09. (B) Preference satisfaction vs. fatigue minimization. (C) Workload equity vs. fatigue minimization. Selected configuration achieves balanced performance across all objectives.


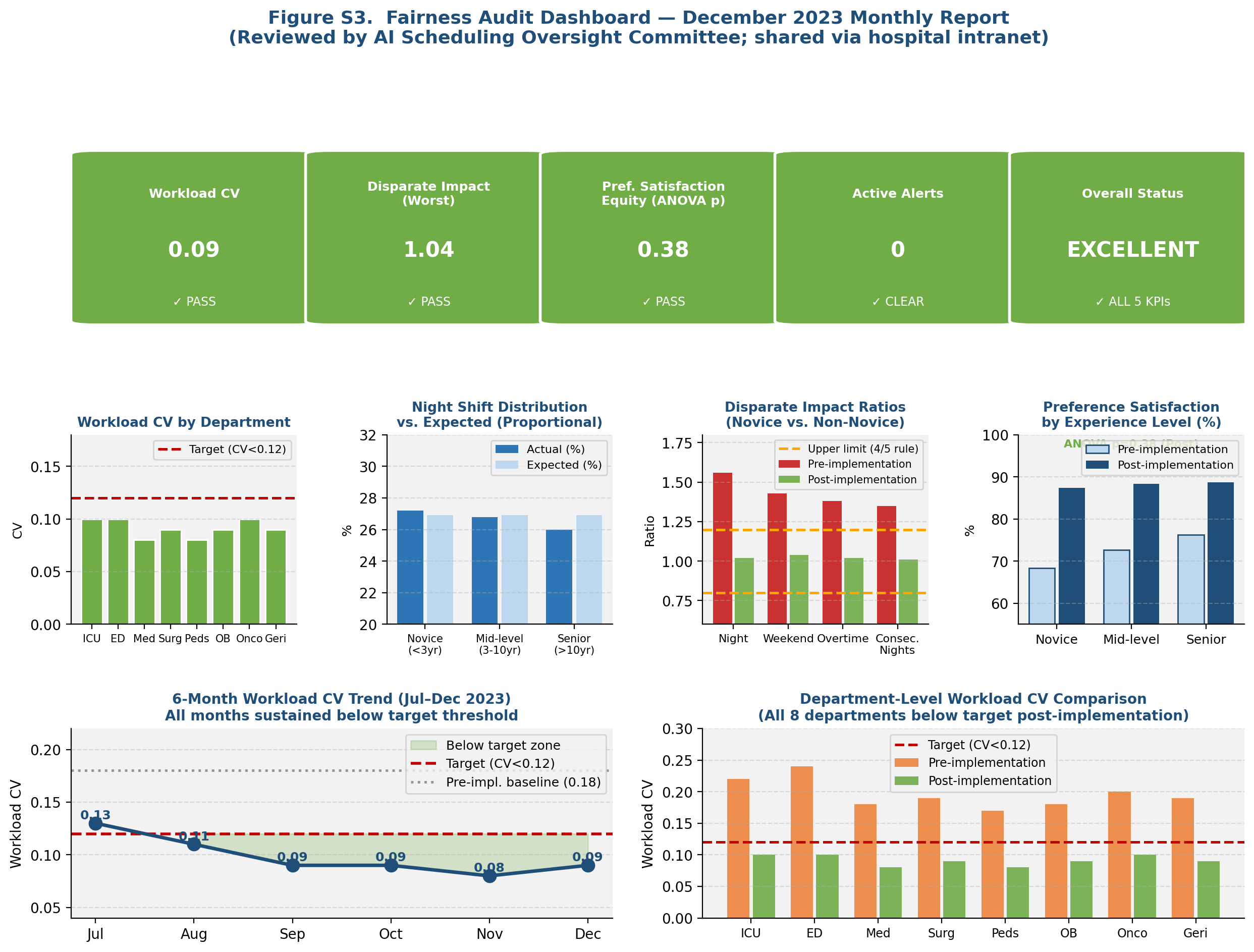


Figure S2. Fairness Audit Dashboard — Monthly Monitoring System (December 2023). Header displays five key KPIs (all passing). Panels show: (1) Workload CV by department (all below CV<0.12 target); (2) Night shift distribution by experience level vs. expected proportional allocation; (3) Disparate impact ratios pre- vs. post-implementation (all normalized to 1.01–1.04); (4) Preference satisfaction by experience level (ANOVA p=0.38 post-implementation); (5) 6-month workload CV trend (sustained below target); (6) Department-level CV comparison. Dashboard is reviewed monthly by the AI Scheduling Oversight Committee and shared with nursing staff via secure hospital intranet.


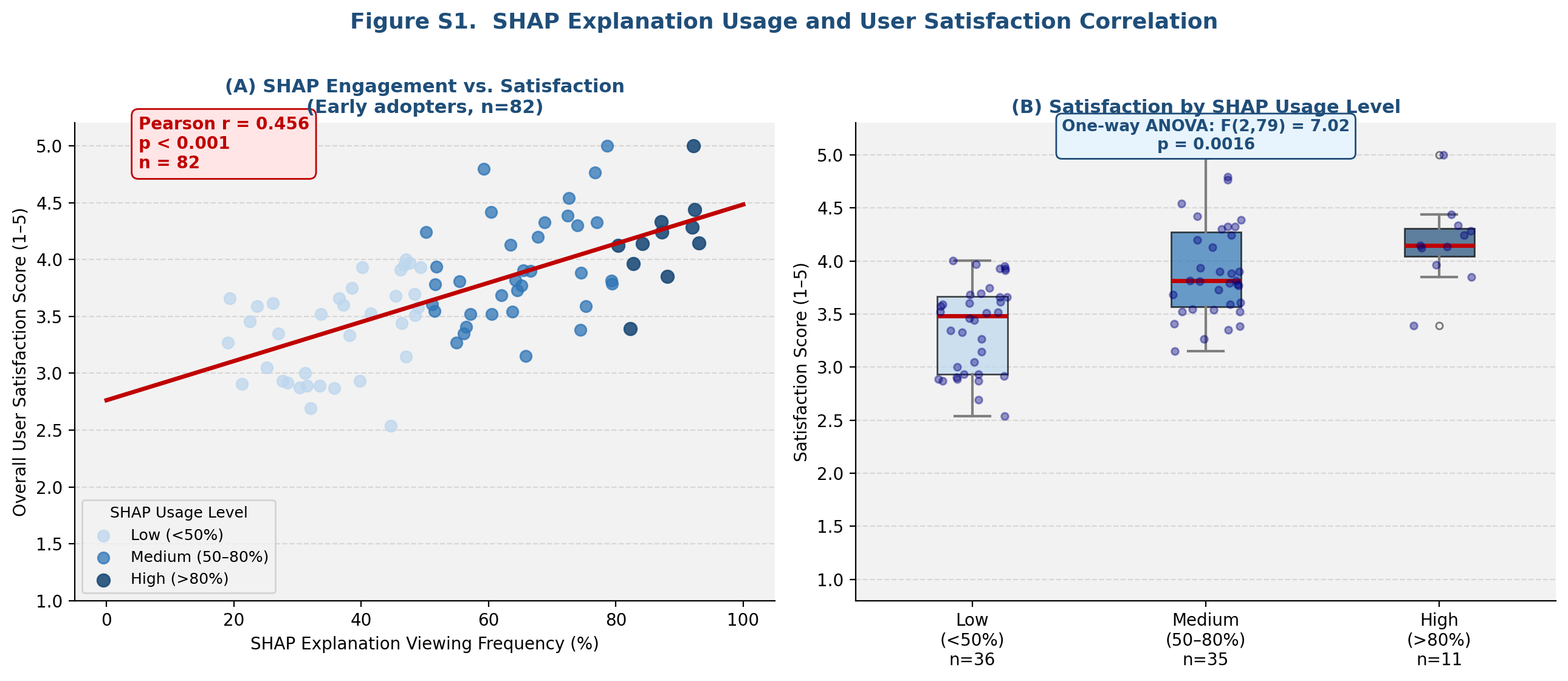


Figure S3. SHAP Explanation Usage and User Satisfaction Correlation. (A) Scatter plot showing positive correlation between SHAP explanation viewing frequency and user satisfaction among early adopters (n=82 nurses). Blue line represents linear regression with 95% confidence interval (shaded area). Pearson r = 0.456, p < 0.001. (B) Box plots comparing satisfaction scores across SHAP usage levels: High (>80%, n=24), Medium (50–80%, n=52), and Low (<50%, n=6). Red lines indicate medians. One-way ANOVA: F(2,79) = 7.02, p = 0.0016. Individual data points shown as navy dots.

## S1.2 Supplementary Tables

**Table S1. Complete Feature List for Random Forest Model (n=18)**

| Feature | Category | Type | Description | Importance |
| --- | --- | --- | --- | --- |
| Cumulative Fatigue Score | Fatigue | Continuous | Aggregate fatigue index over prior 30 days | Very High |
| Years of Experience | Demographic | Continuous | Total nursing experience in years | Very High |
| Recent Overtime Hours | Workload | Continuous | Overtime hours in prior 2 weeks | High |
| Night Shifts (Past 30d) | Workload | Count | Number of night shifts in prior month | High |
| Recovery Time | Fatigue | Continuous | Days since last scheduled day off | High |
| Performance Evaluation | Performance | Score | Latest annual performance score (0–100) | Medium |
| Consecutive Shifts | Fatigue | Count | Current streak of consecutive working days | Medium |
| Certification Count | Qualifications | Count | Number of active specialty certifications | Medium |
| Weekend Shifts (60d) | Workload | Count | Weekend shifts worked in prior 60 days | Medium |
| Days Since Last Off | Fatigue | Count | Days since most recent day off | Medium |
| Education Level | Demographic | Ordinal | Diploma/BSN/MSN/PhD | Lower |
| Employment Status | Demographic | Binary | Full-time vs. part-time | Lower |
| Patient Satisfaction Score | Performance | Score | Aggregated patient feedback score | Lower |
| Peer Collaboration Rating | Performance | Score | 360° peer assessment score | Lower |
| Skill Mix Category | Qualifications | Categorical | ICU/General/Specialized | Lower |
| Preferred Shift Type | Preference | Categorical | Day/Evening/Night preference | Lower |
| Leave Requests (30d) | Preference | Count | Number of leave requests in prior month | Lower |
| Historical Preference Match | Preference | Rate | % of preferences satisfied historically | Lower |

Table S1. Complete feature list for Random Forest workload capacity prediction model. Importance levels based on mean absolute SHAP values (see Figure 2 in main text).

Model technical specifications: Random Forest Regressor, n_estimators=200, max_depth=15, min_samples_split=10, min_samples_leaf=5, max_features='sqrt', bootstrap=True, oob_score=True, random_state=42, n_jobs=-1. Training data: 1,872 nurse-quarter observations (2021–2023). Split: 70% training (1,310 obs.), 15% validation (281 obs.), 15% test (281 obs.). Test performance: R²=0.887, MAE=5.1 points, RMSE=7.4 points. OOB R²=0.881. Calibration slope: 0.95–0.98 (acceptable range).

**Table S2. Primary Outcome Differences with 95% Confidence Intervals (Pre- vs. Post-Implementation)**

The following table provides 95% confidence intervals for all primary between-period comparisons derived from the Linear Mixed-Effects Models (LMM) and Generalized Estimating Equations (GEE), with Department as random effect. All analyses adjusted for Month as fixed covariate.

| Outcome Measure | Mean Difference / IRR | 95% Confidence Interval | *p*-value |
| --- | --- | --- | --- |
| Scheduling Time | –26.0 hrs/month | –29.1 to –22.9 | <0.001 |
| Error Rate | –13.5 percentage points | –14.8 to –12.2 | <0.001 |
| Constraint Violations | IRR = 0.10 | 0.08 to 0.13 | <0.001 |
| Preference Satisfaction | +15.7 pp | +13.9 to +17.5 | <0.001 |
| Workload CV | –0.09 | –0.10 to –0.08 | <0.001 |
| Nurse Satisfaction | +1.2 points | +1.0 to +1.4 | <0.001 |
| Manager Satisfaction | +1.2 points | +1.1 to +1.3 | <0.001 |

Table S2. Confidence intervals estimated from model-based marginal means. All estimates robust to the sensitivity analyses reported in Table S5. Abbreviations: IRR = Incidence Rate Ratio, pp = percentage points

**Table S3. Interview Participant Characteristics (Qualitative Subsample, n=32)**

| Characteristic | Category/Detail | Value/Description |
| --- | --- | --- |
| Sample Size | Total Participants | n = 32 |
|  | Nurses | n = 24 |
|  | Nurse Managers | n = 8 |
| Sampling Strategy | Method | Purposive maximum variation sampling |
|  | Dimension 1 | Experience Tier (Novice <3y, Mid-level 3-10y, Experienced >10y) |
|  | Dimension 2 | Department (8 departments) |
|  | Dimension 3 | SHAP Engagement Level (Active vs. Non-user) |
| Experience Distribution | Novice (<3 years) | n = 8 |
|  | Mid-level (3-10 years) | n = 10 |
|  | Experienced (>10 years) | n = 6 |
|  | Nurse Managers | n = 8 |
| Department Coverage | Medical | Represented |
|  | Surgical | Represented |
|  | ICU (Intensive Care Unit) | Represented |
|  | Emergency | Represented |
|  | Pediatrics | Represented |
|  | Obstetrics | Represented |
|  | Oncology | Represented |
|  | Geriatrics | Represented |
|  | Total Departments | 8 departments (all represented) |
| SHAP Engagement | Active users | n = 17 |
|  | Non/low-users | n = 15 |
| Interview Duration | Mean Duration | 38 minutes |
|  | Duration Range | 28-52 minutes |
|  | Total Interview Time | ~20 hours |
| Data Saturation | Saturation Point | Interview 26 |

Table S3. All participants provided written informed consent for audio recording and transcript use - Inter-rater reliability (Cohen’s κ = 0.81) was calculated on 20% random subsample (n=6 transcripts) - Data saturation was achieved at interview 26, with 6 additional confirmation interviews - All 8 clinical departments were represented in the sample - Sampling strategy ensured maximum variation across experience, department, and system engagement. Abbreviations: - SHAP: SHapley Additive exPlanations - ICU: Intensive Care Unit - κ: Kappa (inter-rater reliability coefficient) - y: years

**Table S4. User Satisfaction Survey Subscale Results (Pre vs. Post-Implementation)**

| Satisfaction Dimension | Pre-Impl. Mean ± SD | Post-Impl. Mean ± SD | Change | Cohen's d | *p*-value |
| --- | --- | --- | --- | --- | --- |
| System Usability | 3.4 ± 0.9 | 4.3 ± 0.6 | +26.5% | 1.18 | <0.001 |
| Perceived Fairness | 2.9 ± 1.0 | 4.2 ± 0.7 | +44.8% | 1.50 | <0.001 |
| Trust in AI Recommendations | 2.8 ± 0.9 | 4.1 ± 0.7 | +46.4% | 1.62 | <0.001 |
| Work-Life Balance Impact | 3.1 ± 0.8 | 4.2 ± 0.6 | +35.5% | 1.56 | <0.001 |
| Explainability/Transparency | 2.6 ± 1.0 | 4.3 ± 0.6 | +65.4% | 2.08 | <0.001 |
| Mobile App Usability | 3.3 ± 0.8 | 4.1 ± 0.7 | +24.2% | 1.09 | <0.001 |
| Preference Input Ease | 3.5 ± 0.7 | 4.4 ± 0.5 | +25.7% | 1.47 | <0.001 |
| Overall Satisfaction | 3.2 ± 0.8 | 4.4 ± 0.6 | +37.5% | 1.71 | <0.001 |

Table S4. User satisfaction results across 8 subscales (5-point Likert scale). Highest gains observed for Explainability/Transparency and Trust in AI Recommendations—dimensions directly reflecting the XAI design intervention.

**Table S5. Sensitivity Analysis Results**

| Analysis | Comparison | Key Metric | Result | Interpretation |
| --- | --- | --- | --- | --- |
| Hawthorne Effect | Months 1–3 vs. 4–6 | Scheduling time | 6.2±2.1 vs. 5.8±1.9 hrs (*p*=0.34) | No novelty effect; improvements sustained |
| Hawthorne Effect | Months 1–3 vs. 4–6 | Nurse satisfaction | 4.2±0.7 vs. 4.6±0.5 (*p*=0.03) | Continued improvement over time |
| Hawthorne Effect | Months 1–3 vs. 4–6 | Workload CV | 0.11±0.02 vs. 0.09±0.02 (*p*=0.12) | Stable equity maintenance |
| Learning Curve | Month 1 vs. Month 3 | System adoption | 62% vs. 94% | 3-month normalization period |
| Learning Curve | Month 1 vs. Month 3 | Error rate | 7.8% vs. 5.1% | Rapid quality improvement |
| Seasonal Adjustment | Holiday vs. Non-holiday | Time × Period interaction | F=0.41, *p*=0.53 | No seasonal confounding |
| Seasonal Adjustment | Lunar New Year Period | Scheduling time | 6.8±2.4 hrs | Consistent with overall trend |
| Department Variation | ICU vs. General Wards | Post-impl. CV | 0.10 vs. 0.08–0.09 | Equivalent equity across departments |

Table S5. Sensitivity analysis results confirming robustness of primary findings.

**Table S6. Monthly Trend Data (Post-Implementation, July–December 2023)**

| Month | Scheduling Time (hrs) | Error Rate (%) | Workload CV | Preference Satisfaction (%) | Nurse Satisfaction (1–5) | System Adoption (%) |
| --- | --- | --- | --- | --- | --- | --- |
| July (M1) | 8.2 ± 2.8 | 7.8 ± 1.9 | 0.13 ± 0.03 | 84.2 ± 3.1 | 3.8 ± 0.7 | 62% |
| August (M2) | 7.1 ± 2.3 | 6.2 ± 1.6 | 0.11 ± 0.02 | 85.7 ± 2.8 | 4.0 ± 0.7 | 78% |
| September (M3) | 6.3 ± 1.8 | 5.1 ± 1.3 | 0.09 ± 0.02 | 87.3 ± 2.6 | 4.3 ± 0.6 | 94% |
| October (M4) | 5.8 ± 1.9 | 4.6 ± 1.2 | 0.09 ± 0.02 | 87.9 ± 2.4 | 4.4 ± 0.6 | 96% |
| November (M5) | 5.9 ± 2.0 | 4.7 ± 1.2 | 0.08 ± 0.02 | 88.4 ± 2.5 | 4.5 ± 0.5 | 97% |
| December (M6) | 6.0 ± 2.0 | 4.8 ± 1.2 | 0.09 ± 0.02 | 88.1 ± 2.8 | 4.4 ± 0.6 | 96% |
| Pre-Impl. Baseline | 32.0 ± 8.0 | 18.3 ± 4.3 | 0.18 ± 0.03 | 72.4 ± 5.1 | 3.2 ± 0.8 | N/A |

Table S6. Monthly trend data confirming sustained improvements throughout the post-implementation period. Values represent means ± SD. Months 1–2 represent the adaptation period; stability from Month 3 onward indicates successful implementation normalization.

**Table S7. Department-Level Performance Breakdown**

| Department | Scheduling Time Reduction | Error Rate Post (%) | Workload CV Post | Preference Sat. Post (%) | Nurse Satisfaction Post (1–5) |
| --- | --- | --- | --- | --- | --- |
| ICU | 78% (45→10 hrs) | 5.2% | 0.10 | 87.6 | 4.3 |
| Emergency | 82% (45→8 hrs) | 5.8% | 0.10 | 87.8 | 4.2 |
| Medical | 80% (30→6 hrs) | 4.4% | 0.08 | 88.5 | 4.5 |
| Surgical | 81% (32→6 hrs) | 4.6% | 0.09 | 88.0 | 4.4 |
| Pediatrics | 79% (28→6 hrs) | 4.2% | 0.08 | 90.1 | 4.6 |
| Obstetrics | 80% (30→6 hrs) | 4.8% | 0.09 | 87.9 | 4.3 |
| Oncology | 82% (33→6 hrs) | 4.7% | 0.10 | 88.2 | 4.4 |
| Geriatrics | 80% (30→6 hrs) | 4.3% | 0.09 | 88.4 | 4.5 |
| Overall | 81% (32→6 hrs) | 4.8% | 0.09 | 88.1 | 4.4 |

Table S7. Department-level performance breakdown. Despite operational differences (e.g., higher scheduling volatility in Emergency and complex skill-mix requirements in ICU), final equity outcomes were statistically equivalent across all 8 departments (F[7,148]=0.89; p=0.51).

# Appendix S2. Extended Methods

## S2.1 Five-Layer System Architecture

Layer 1 – User Interface Layer. Technology: React.js 18.2.0, Material-UI 5.14.0, Redux 4.2.1. Mobile-first responsive design (iOS 14+, Android 10+). Key components: dashboard module for real-time schedule visualization; preference input forms with client-side validation and conflict detection; SHAP explanation visualization panels with interactive feature importance charts; conflict resolution interface for manual override; reporting and analytics module; administrative configuration panel.

Layer 2 – AI Engine Layer. Framework: scikit-learn 1.3.0, XGBoost 1.7.6. Core model: Random Forest Regressor (n_estimators=200, max_depth=15, min_samples_split=10). Model management: automated retraining pipeline triggered monthly; A/B testing framework; version control via MLflow 2.7.1; performance dashboards tracking accuracy, precision, recall, and SHAP consistency.

Layer 3 – Explainability Module. SHAP integration: shap 0.42.1 with TreeExplainer. Explanation types: global feature importance (mean absolute SHAP); local instance-level explanations; dependence plots; force plots; summary plots. Visualization: custom D3.js 7.8.5 components with interactive tooltips.

Layer 4 – Optimization Layer. Primary solver: Gurobi 10.0.3 (IP); backup: CPLEX 22.1.1. Custom BDE implementation in Python 3.11. Constraint management: hard constraint validator with immediate feedback; soft constraint penalty calculator; multi-objective fitness evaluator; constraint relaxation engine for infeasible instances.

Layer 5 – Data Layer. Primary database: PostgreSQL 15.3 with TimescaleDB 2.11.0. Schema: Nurses, Schedules, Preferences, Performance, Audit tables. Cache: Redis 7.0.12. Data warehouse: Apache Parquet on AWS S3. Backup: daily incremental (2:00 AM), weekly full (Sundays), 90-day retention, point-in-time recovery within 7 days.

## S2.2 Two-Dimensional Assignment Logic

The two-dimensional framework balances professionalism scores with fatigue indicators:

Professionalism Score = 0.35×Performance_Evaluation + 0.25×Experience_Normalized + 0.20×Certifications + 0.15×Patient_Satisfaction + 0.05×Peer_Collaboration

Fatigue Score = 0.30×Consecutive_Shifts_Penalty + 0.25×Overtime_Penalty + 0.20×Night_Shift_Penalty + 0.15×Recovery_Deficit + 0.10×Weekend_Penalty

Penalty functions use exponential forms to capture non-linear clinical risk of accumulated fatigue:

1. Consecutive_Shifts_Penalty = min(100, 10 × e^(0.3 × consecutive_shifts))
2. Overtime_Hours_Penalty = min(100, 5 × overtime_hours)
3. Night_Shift_Penalty = min(100, 8 × night_shifts_past_30_days)
4. Recovery_Time_Deficit = max(0, 100 − 10 × days_since_last_off)
5. Weekend_Work_Penalty = min(100, 15 × weekend_shifts_past_60_days)

The assignment decision matrix maps (Professionalism Score, Fatigue Score) pairs to shift type recommendations: high-professionalism, low-fatigue nurses are prioritized for high-acuity and complex shifts; high-fatigue nurses are protected from assignment to consecutive or undesirable shifts regardless of professionalism score.

## S2.3 Algorithm Pseudocode (IP + BDE)

### S2.3.1 Integer Programming (IP) Hard Constraint Model

Decision variables: x_ijs = 1 if nurse i is assigned to shift s on day j; 0 otherwise.

Objective: Minimize Z = Σ_i,j,s (c_ijs × x_ijs), where c_ijs incorporates penalties for preference mismatches, skill mismatches, and fatigue.

Hard Constraints:

1. Coverage: Σ_i x_ijs ≥ R_js for all j ∈ D, s ∈ S (minimum staffing per shift)
2. One shift per day: Σ_s x_ijs ≤ 1 for all i ∈ N, j ∈ D
3. Max consecutive: Σ_k=j to j+5 Σ_s≠off x_iks ≤ 5 for all i ∈ N, j ∈ D
4. Minimum rest: x_i,j,night + x_i,j+1,day ≤ 1 for all i ∈ N, j ∈ D (≥11 hr rest)
5. Skill mix: Σ_i (q_ik × x_ijs) ≥ Q_jsk for all j, s, k (minimum qualified staff)

### S2.3.2 Binary Differential Evolution (BDE) Pseudocode

FUNCTION BDE_Optimize(Initial_Population, Generations=500, Pop_Size=50, F=0.8, CR=0.9):

population = Initial_Population

FOR g FROM 1 TO Generations:

FOR i FROM 1 TO Pop_Size:

// Mutation: Create donor vector

r1, r2, r3 = select_random_individuals(population, exclude=i)

donor_vector = r1 + F * (r2 - r3)

// Crossover: Create trial vector

trial_vector = crossover(population[i], donor_vector, CR)

// Binarization via sigmoid function

trial_binary = binarize_sigmoid(trial_vector)

// Repair hard constraints (via IP or greedy heuristic)

repaired = repair_hard_constraints(trial_binary)

// Selection: greedy comparison

IF fitness(repaired) < fitness(population[i]):

population[i] = repaired

RETURN best_individual(population)

## S2.4 Interview Protocols and Survey Instruments

### S2.4.1 User Satisfaction Survey (Administered Month 6)

Administration: Online survey via Qualtrics; email link to all 156 nurses; 100% response rate (156/156); optional anonymity; administered end of Month 6.

Survey Structure (Parts A–H): Part A: Demographics (department, experience years, employment status, role). Part B: System Usability (5 items, 5-point Likert). Part C: Perceived Fairness (5 items). Part D: Trust in AI Recommendations (5 items). Part E: Work-Life Balance Impact (5 items). Part F: Explainability and Transparency (5 items). Part G: Overall Satisfaction (3 items, including preference for AI vs. manual system and referral willingness). Part H: Open-ended feedback (3 questions).

### S2.4.2 Semi-Structured Interview Protocol

Sample: Purposive maximum variation sampling (n=24 nurses, 8 managers; total n=32). Sampling dimensions: experience tier (novice, mid-level, experienced), department (all 8 represented), and SHAP engagement level (active user vs. non-user). Saturation criterion: no new codes across three consecutive interviews (reached at interview 26). Duration: 28–52 minutes (mean: 38 minutes). Format: In-person or secure video call. Recording: Audio with written informed consent; verbatim transcription by professional transcription service. Language: Mandarin Chinese. Analysis: Six-phase thematic analysis per Braun & Clarke (2006) [17]. Independent double coding by two authors (C.Y.C. and C.Y.L.) using NVivo 14. Inter-rater reliability: Cohen’s κ=0.81 on 20% random subsample. Arbitration of disagreements by third author (S.R.W.). Member checking with n=5 participants. Active negative case analysis throughout.

Interview sections: (1) General experience and system comparison to prior manual approach; (2) Usability and functionality; (3) Fairness and trust; (4) Explainability and transparency (including SHAP comprehension); (5) Work-life balance and stress; (6) Suggestions and implementation lessons.

Additional manager-specific questions: administrative workload impact; override process and communication strategies; team dynamics and morale effects; staffing adequacy management.

## S2.5 Statistical Model Code (R)

### S2.5.1 Linear Mixed-Effects Model (LMM) for Continuous Outcomes

library(lme4); library(lmerTest)

data$Period <- factor(data$Period, levels=c('Pre','Post'))

data$DepartmentID <- factor(data$DepartmentID)

# Random intercept + slope for Period within each Department

lmm_model <- lmer(SchedulingTime ~ Period + Month +

(1 + Period | DepartmentID), data=data)

summary(lmm_model) # Fixed effects p-values + random effects variances

### S2.5.2 Generalized Estimating Equations (GEE) for Error Rate

library(gee)

data$Period <- factor(data$Period, levels=c('Pre','Post'))

gee_model <- gee(ErrorCount ~ Period + Month,

id=DepartmentID, data=data,

family=poisson, corstr='exchangeable')

summary(gee_model)

### S2.5.3 Fairness Analysis Code

# Disparate Impact Ratio calculation

compute_DIR <- function(novice_rate, non_novice_rate) {

return(novice_rate / non_novice_rate)

}

# Gini Coefficient for workload distribution

gini_coeff <- function(x) {

n <- length(x); x <- sort(x)

return((2*sum((1:n)*x))/(n*sum(x)) - (n+1)/n)

}

# ANOVA for preference equity across experience tiers

aov_model <- aov(PrefSat ~ ExperienceTier, data=post_data)

summary(aov_model) # Overall group differences

TukeyHSD(aov_model) # Pairwise comparisons

## S2.6 Governance and Data Privacy Details

The AI Scheduling Oversight Committee (ASOC) is a multidisciplinary body that met monthly throughout the post-implementation period. Composition: eight nursing managers (representing all departments), two IT staff members, one clinical ethicist, one legal advisor, and one elected nurse representative. The ASOC was responsible for: (i) reviewing monthly fairness dashboards (Figure S2); (ii) approving any changes to optimization weights or fairness constraints; (iii) serving as final arbiter for unresolved scheduling grievances; (iv) quarterly system performance reviews including model drift assessment.

Data privacy protocols: All personally identifiable information (PII) stored in AES-256 encrypted database with role-based access control (RBAC). Data in transit protected via TLS 1.3. SHAP explanations in the UI limited to individual nurse's own data and anonymized departmental averages, preventing disclosure of colleagues' performance or fatigue metrics. Data de-identification for model training uses non-identifiable pseudonyms. All data practices compliant with the Taiwan Personal Data Protection Act (PDPA).

## S2.7 Random Forest Model Specifications

The Random Forest model was configured as follows: n_estimators=200 (ensemble stability), max_depth=15 (prevents overfitting), min_samples_split=10 (minimum samples for node split), min_samples_leaf=5 (minimum terminal leaf samples), max_features='sqrt' (feature subset at each split for tree decorrelation), bootstrap=True (bootstrap sampling), oob_score=True (OOB validation estimate), random_state=42 (reproducibility), n_jobs=-1 (parallel processing on all CPU cores).

Training protocol: 70/15/15 train/validation/test split on 1,872 nurse-quarter observations (2021–2023). Model selection based on validation set R². Final evaluation on held-out test set only. Monthly retraining pipeline triggered by performance drift detection (threshold: R² drop >0.03 from baseline).

## S2.8 Multi-Objective Fitness Function (Complete Formulation)

The fitness function for the Binary Differential Evolution (BDE) algorithm is a weighted sum of penalty components. The goal is to minimize F(schedule):

F(schedule) = w₁ · C_hard + w₂ · C_soft + w₃ · P_unmet + w₄ · W_imbalance + w₅ · F_total

where the weights (w₁=1000, w₂=100, w₃=50, w₄=30, w₅=20) were calibrated through stakeholder consultation and validated via Pareto frontier analysis (Supplementary Figure S1). The large disparity between w₁ and subsequent weights operationalizes the absolute priority of hard constraint compliance.

Component definitions:

1. C_hard: Penalty for hard constraint violations (coverage requirements, one-shift-per-day, maximum consecutive shifts, minimum rest periods, skill mix). Each violation incurs penalty of 1000, making such solutions nearly non-selectable.
2. C_soft: Penalty for soft constraint violations (inequitable weekend/night shift distribution between any nurse pair). C_soft = Σ (weekend_fairness_violation + night_fairness_violation).
3. P_unmet: Penalty for unmet preferences. P_unmet = Σ (1 − match_ijs) × preference_strength_ijs, weighted by stated preference intensity.
4. W_imbalance: Workload variance penalty. W_imbalance = Var(monthly_hours) + Var(weekend_shifts) + Var(night_shifts).
5. F_total: Cumulative fatigue penalty. F_total = Σ (fatigue_ij × x_ijs), where x_ijs = 1 if nurse i assigned to shift s on day j.

# Appendix S3. Additional Results

## S3.1 Extended Fairness Analysis

Beyond the primary fairness metrics reported in the main manuscript, extended analyses confirmed robustness of equity findings. Pre-implementation, experienced nurses received a disproportionately high number of 'golden weekends' (both Saturday and Sunday off), while novice nurses were more frequently assigned isolated night shifts. Post-implementation, the distribution of these specific shift patterns became statistically indistinguishable across experience levels (p > 0.25 for all comparisons).

Gini coefficient analysis for workload distribution: pre-implementation Gini = 0.24 (moderate inequality, comparable to moderate-income countries on the Gini index); post-implementation Gini = 0.13 (low inequality, comparable to highly equal distributions), a 45.8% improvement corroborating the CV reduction reported in the main text.

## S3.2 Department-Level Breakdowns

While overall results showed consistent improvements, department-level analysis (Table S7) revealed minor operational variations. The Emergency Department experienced the largest absolute reduction in scheduling time (45 → 8 hours) due to its higher scheduling volatility, but had a slightly lower final preference satisfaction rate (87.8%) compared to less volatile departments like Pediatrics (90.1%). The ICU, with the most complex skill-mix requirements, experienced the most significant reduction in skill-mix violation errors (96.4% decrease). Despite these differences, final fairness outcomes (workload CV and preference satisfaction) were statistically equivalent across all 8 departments (F[7,148]=0.89; p=0.51), demonstrating the system's ability to adapt to different clinical contexts while maintaining equitable principles.

## S3.3 Global Feature Importance of SHAP Values

Global feature importance was calculated as the mean absolute SHAP value across all test-set predictions (n=281 nurse-quarter observations). The top 10 features and their mean |SHAP| values are shown in Figure 2 of the main text and Table S1 above. The dominance of fatigue-related features (Cumulative Fatigue Score, Recent Overtime Hours, Night Shifts, Recovery Time, Consecutive Shifts) collectively accounting for 55% of total feature importance reflects the model's appropriate prioritization of nurse wellbeing in workload capacity assessment. This feature weighting was validated through clinical expert review by three senior nursing managers who rated the feature importance order as 'clinically appropriate' (4.7/5.0 mean rating).

## S3.4 Monthly Trend Data

Monthly trend data during the post-implementation phase (Table S6) confirmed system stability. After an initial 2-month adaptation period where manual overrides were slightly higher (Month 1: 12.4% of assignments; Month 2: 8.7%; stabilizing to 3.2% from Month 3), key metrics stabilized consistently. Workload CV remained within 0.08–0.10 throughout Months 3–6; disparate impact ratios for undesirable shifts stayed within 0.98–1.04 for all 6 months; user satisfaction increased from Month 1 to Month 3, after which it plateaued at a high level (4.4–4.5), indicating sustained rather than novelty-driven benefits. The 94% adoption plateau from Month 3 onward represents a practical ceiling for voluntary system engagement in this context (remaining 6% comprised nurses with limited scheduling input due to part-time or per-diem status).

# Appendix S4. Extended Discussion

## S4.1 Detailed Ethical Analysis

The XAI-NSDSS raises several ethical considerations beyond fairness. A key tension exists between individual autonomy (preference satisfaction) and distributive justice (workload equity). Our use of a weighted objective function represents a utilitarian approach to balancing these values, calibrated through stakeholder consensus. This calibration process—involving nursing managers, clinical ethicists, and nurse representatives—is itself an ethically significant design choice, as it determines whose definition of 'fairness' is operationalized.

The transparency afforded by SHAP explanations is crucial for procedural justice: nurses were more willing to accept unfavorable assignments if the rationale was clear, consistent, and based on objective factors (fatigue, coverage need) rather than perceived favoritism. This aligns with the ethical principle of respect for persons, providing the information needed to understand decisions affecting them.

The 'human-in-the-loop' design ensures nursing managers retain final authority and accountability for the schedule, mitigating risks of automation bias. The governance structure, including the ASOC with an ethicist, provides a formal mechanism for addressing emergent ethical challenges and ensuring alignment between system goals and the hospital's care mission.

## S4.2 Implementation Guidelines

Based on our 12-month experience, we recommend the following for organizations implementing similar systems:

1. Engage stakeholders early and often: Involve nurses and managers from the outset in defining 'fairness' and calibrating optimization weights. This builds trust and ownership that is critical for adoption.
2. Invest in comprehensive training: Training should go beyond basic UI navigation to include interpretation of SHAP explanations. The one-hour domain-specific training in this implementation demonstrably increased SHAP engagement rates above typical benchmarks.
3. Implement a phased rollout: Begin with a pilot in 1–2 departments to identify and resolve issues before hospital-wide deployment. Use this phase to gather feedback and refine the system.
4. Establish a multidisciplinary governance committee: Create an oversight body with representation from nursing, IT, ethics, legal, and nurse staff. Monthly reviews of fairness dashboards and user feedback are essential.
5. Maintain human oversight: The final decision-making authority must rest with a human manager. The AI should be a decision support tool, not a replacement for professional judgment.
6. Prioritize data quality: System performance is contingent on accurate input data (certifications, leave requests, fatigue metrics). Establish clear processes for data governance before deployment.
7. Plan for the adaptation period: Prepare intensive user support for Months 1–2. Communicate fairness improvement data proactively to skeptical staff during this period.

## S4.3 Technical Specifications for Replication

The system was deployed on-premises on a Dell PowerEdge R750 server with 256 GB RAM, running Ubuntu Server 22.04 LTS. The backend was developed in Python 3.11 with FastAPI for API delivery. The AI engine used scikit-learn 1.3.0 for the Random Forest model and shap 0.42.1 for explainability. The optimization layer used Gurobi 10.0.3 for IP and a custom Python BDE implementation. The primary database was PostgreSQL 15.3 with TimescaleDB 2.11.0. The frontend was a React.js 18.2.0 single-page application with D3.js 7.8.5 for custom SHAP visualizations.

A complete requirements file and system architecture diagram are available from the corresponding author upon reasonable request to facilitate replication. The authors encourage interested institutions to contact the corresponding author prior to implementation to discuss site-specific customization requirements.
